# Fabrication and Characterization of a PZT-Based Touch Sensor Using Combined Spin-Coating and Sputtering Methods

**DOI:** 10.3390/s25133938

**Published:** 2025-06-24

**Authors:** Melih Ozden, Omer Coban, Tevhit Karacali

**Affiliations:** 1 Department of Electrical and Electronics Engineering, Erzincan Binali Yildirim University, 24100 Erzincan, Türkiye; melih.ozden@erzincan.edu.tr; 2 Department of Electrical and Electronics Engineering, Ataturk University, 25240 Erzurum, Türkiye; omercoban@atauni.edu.tr

**Keywords:** lead zirconate titanate (PZT), spin-coating, RF sputtering, hybrid deposition, touch sensor, BVD model, impedance

## Abstract

This study presents the successful fabrication of lead zirconate titanate (PZT) thin films on silicon (Si) substrates using a hybrid deposition method combining spin-coating and RF sputtering techniques. Initially, a PZT layer was deposited through four successive spin-coating cycles, followed by an additional layer formed via RF sputtering. The resulting multilayer structure was annealed at ${700\;}^{{}^{\circ}}$C for 2 h to improve crystallinity. Comprehensive material characterization was conducted using XRD, SEM, cross-sectional SEM, EDX, and UV–VIS absorbance spectroscopy. The analyses confirmed the formation of a well-crystallized perovskite phase, a uniform surface morphology, and an optical band gap of approximately 3.55 eV, supporting its suitability for sensing applications. Building upon these findings, a multilayer PZT-based touch sensor was fabricated and electrically characterized. Low-frequency I–V measurements demonstrated consistent and repeatable polarization behavior under cyclic loading conditions. In addition, |Z|–f measurements were performed to assess the sensor’s dynamic electrical behavior. Although expected dielectric responses were observed, the absence of distinct anti-resonance peaks suggested non-idealities linked to ${\mathrm{Ag}}^{+}$ ion diffusion from the electrode layers. To account for these effects, the classical Butterworth–Van Dyke (BVD) equivalent circuit model was extended with additional inductive and resistive components representing parasitic pathways. This modified model provided excellent agreement with the measured impedance and phase data, offering deeper insight into the interplay between material degradation and electrical performance. Overall, the developed sensor structure exhibits strong potential for use in piezoelectric sensing applications, particularly for tactile and pressure-based interfaces.

## 1. Introduction

Lead zirconate titanate (PZT), chemically represented as ${\mathrm{Pb}[\mathrm{Zr}}_{x}{\mathrm{Ti}}_{1-x}{]O}_{3}$, (0≤x≤1), is a multifunctional ceramic material extensively utilized in advanced technological applications due to its remarkable dielectric, piezoelectric, pyroelectric, and ferroelectric properties [1]. The combination of high dielectric permittivity and excellent mechanical robustness renders PZT suitable for diverse applications, ranging from sensing systems to energy harvesting devices [2]. The piezoelectric nature of PZT enables the direct transduction of mechanical stress into electrical signals. In parallel, its intrinsic ferroelectricity supports stable polarization under the effect of external electric fields, making it indispensable for high-performance electronic and electromechanical systems. Furthermore, the pyroelectric properties of PZT enable it to effectively detect temperature variations, thus positioning it as a crucial material in thermal sensing technologies [3].

Owing to these exceptional attributes, PZT has emerged as an essential material in a wide array of technologies, including ultrasonic transducers [4,5], infrared detectors [6,7], electro-optical components [8], actuators [9,10], modulators [11], piezoelectric resonators [12,13], multilayer ceramic capacitors (MLCs) [14], microelectromechanical systems (MEMSs) [15], energy harvesting platforms [16], and biomedical sensors [17]. Consequently, the importance and versatility of PZT in contemporary electronic and electromechanical systems continue to expand significantly.

A wide range of deposition techniques has been introduced to produce high-quality PZT films that can effectively harness these features. These techniques can be broadly classified into three principal categories: physical, chemical, and sol–gel-based deposition methods. Physical methods include physical vapor deposition (PVD) [18], electron beam evaporation (e-beam) [19], sputtering [20], pulsed laser deposition (PLD) [21], and molecular beam epitaxy (MBE) [22]. Chemical approaches encompass chemical vapor deposition (CVD) [23], atomic layer deposition (ALD) [24], and chemical solution deposition (CSD) [25]. Additionally, sol–gel-based deposition techniques such as spin-coating [26,27], spray pyrolysis [28], dip-coating [29], and electrophoretic deposition (EPD) [30] are widely implemented. The choice of deposition method critically affects the crystalline structure, surface morphology, and electrical properties of the resultant PZT films; therefore, selecting an appropriate technique tailored to specific application requirements is essential.

In this work PZT films were deposited onto p-type (100)-oriented silicon substrates through a hybrid deposition strategy, integrating spin-coating and RF sputtering methods. Recent advancements in PZT film deposition have underscored the need to choose suitable deposition methods to guarantee both structural integrity and functional efficacy. In this study, PZT films are created using a hybrid method that combines RF sputtering and spin-coating. This approach was adopted to harness the distinct advantages of each technique while mitigating their respective limitations. Spin-coating was initially used to deposit a seed layer that promotes perovskite phase formation and helps reduce lattice mismatch between the silicon substrate and the active PZT layer. In addition, spin-coating contributed to achieving a baseline film thickness, which was otherwise insufficient when sputtering alone was used. However, spin-coated films often suffer from surface cracking and non-uniform morphology due to drying-induced stress. To reduce these problems, and enhance film density, RF sputtering was subsequently applied to deposit a second PZT layer. This sputtered layer increased overall structural quality. The combination of these two methods provided a more controlled microstructure.

The fabricated films were systematically characterized considering structural, morphological, optical, and electrical properties. Leveraging these experimental insights, a multilayered PZT-based touch sensor was subsequently designed and fabricated. This study thus comprises two primary phases: material development and device implementation. In the first stage of the study, PZT films were deposited onto silicon substrates, followed by a comprehensive set of characterizations including XRD, SEM, cross-sectional SEM, and UV–VIS absorbance spectroscopy. The favorable outcomes understood from these analyses provided the foundation for proceeding to the second stage: device fabrication. For the electrical characterization of the fabricated device (sandwich-like), I–V and Z–f measurements were conducted. In addition, touch response tests were performed to evaluate the sensor’s functionality under mechanical stimulation conditions. The remainder of this article is structured as follows: Section 2 provides comprehensive details of the deposition methods, fabrication processes, and characterization techniques employed. Section 3 discusses the characterization outcomes of the developed PZT films and evaluates the operational performance of the fabricated touch sensor. Finally, Section 4 presents a detailed discussion, highlights key findings, and concludes.

## 2. Materials and Methods

**Figure 1:**
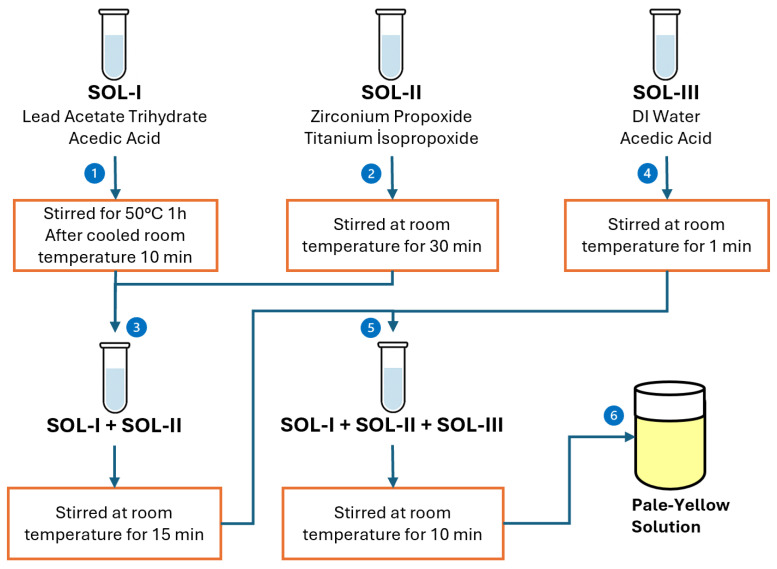
Preparation steps of the final precursor solution used in the spin-coating process for PZT thin film deposition.

Initially, PZT films (Sigma-Aldrich, St. Louis, MO, USA) were deposited using spin-coating, commencing with the formulation of a final precursor solution. In the initial stage, films were deposited by spin-coating, starting with the preparation of a final precursor solution. This solution was obtained by carefully mixing three distinct stock solutions at precisely determined ratios to achieve the target stoichiometry of ${\mathrm{Pb}}_{1}$[${\mathrm{Zr}}_{0.52}$${\mathrm{Ti}}_{0.48}$]$O_{3}$. Figure 1 illustrates the schematic diagram of this solution preparation.

Firstly, SOL-I was prepared by dissolving 3.794 g of lead acetate trihydrate in 5 mL of acetic acid at ${50\;}^{{}^{\circ}}$C, followed by stirring for 1 h. The solution was then allowed to cool to room temperature for 10 min. Separately, SOL-II was prepared by mixing 1.8 mL of zirconium propoxide with 1.4 mL of titanium isopropoxide and stirring the mixture at room temperature for 30 min. After both solutions were ready, SOL-I and SOL-II were combined and stirred at room temperature for 15 min. Subsequently, SOL-III was prepared by mixing 1 mL of deionized water with 0.8 mL of acetic acid and stirring for 1 min. Finally, SOL-III was added dropwise into the pre-mixed SOL-I and SOL-II solution, and the resulting mixture was stirred at room temperature for 10 min. This step-by-step process yields a homogeneous pale-yellow sol suitable for further film deposition, as shown in Figure 1 [31].

**Figure 2:**
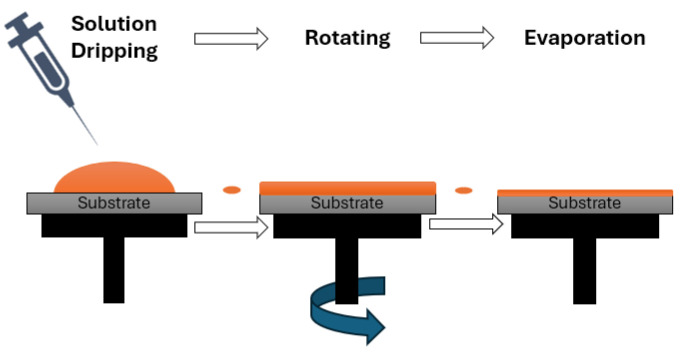
Schematic diagram of the spin-coating process for PZT thin film deposition.

Subsequently, the precursor solution was deposited onto pre-cleaned, p-type (100)-oriented silicon substrates and spin-coated at 3000 rpm for 30 s to ensure uniform film thickness. After each spin-coating cycle, substrates were dried at ${350\;}^{{}^{\circ}}$C for 10 min to remove residual solvents. This spin-coating and drying procedure was repeated four times to achieve the desired total film thickness. Figure 2 provides a schematic representation of the spin-coating process.

**Figure 3:**
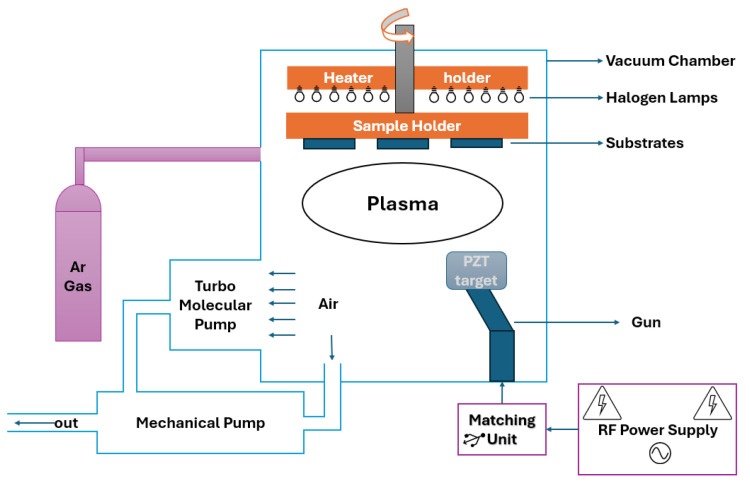
Schematic diagram of the RF sputtering system used for the deposition of the top PZT layer.

In the second stage, an additional PZT layer was deposited via RF sputtering to enhance film quality by utilizing the advantages offered by physical vapor deposition. The RF sputtering system used is schematically illustrated in Figure 3, with detailed deposition parameters summarized in Table 1. Following deposition, films underwent a thermal annealing process at ${700\;}^{{}^{\circ}}$C for 2 h to promote crystallization.

The fabricated films were systematically characterized to evaluate their structural, morphological, compositional, and optical properties. X-ray diffraction (XRD), scanning electron microscopy (SEM), cross-sectional SEM, energy-dispersive X-ray spectroscopy (EDX), and UV–VIS absorbance spectroscopy were employed. XRD analysis was conducted using a Panalytical Empyrean diffractometer equipped with Cu Kα radiation (λ=1.5405 Å), scanning between $20^{{}^{\circ}}$ and $60^{{}^{\circ}}$ (2θ) at a step size of $0.026^{{}^{\circ}}$ over one hour. Surface morphology was investigated using an FEI Quanta FEG 450 SEM (FEI Company, Hillsboro, OR, USA) at magnifications of 5000×, 10,000×, and 30,000×. Elemental composition was confirmed via integrated EDX analysis. Optical absorbance spectra were obtained using a Shimadzu RF-5301 PC (Shimadzu Corporation, Kyoto, Japan) spectrophotometer with a resolution of 1 nm. The designed sensors were evaluated for impedance using an EDC-1630 digital LCR meter (Electronic Development Corporation, Newton, MA, USA) and a PocketVNA vector network analyzer (PocketVNA, Berlin, Germany). Current–voltage (I–V) behavior was characterized using a Keithley 487 picoammeter/voltage source (Keithley Instruments, Cleveland, OH, USA), and the sensor output signals were monitored using a Uni-T UTD2052CL digital oscilloscope (UNI-T, Dongguan, Guangdong, China).

**Figure 4:**
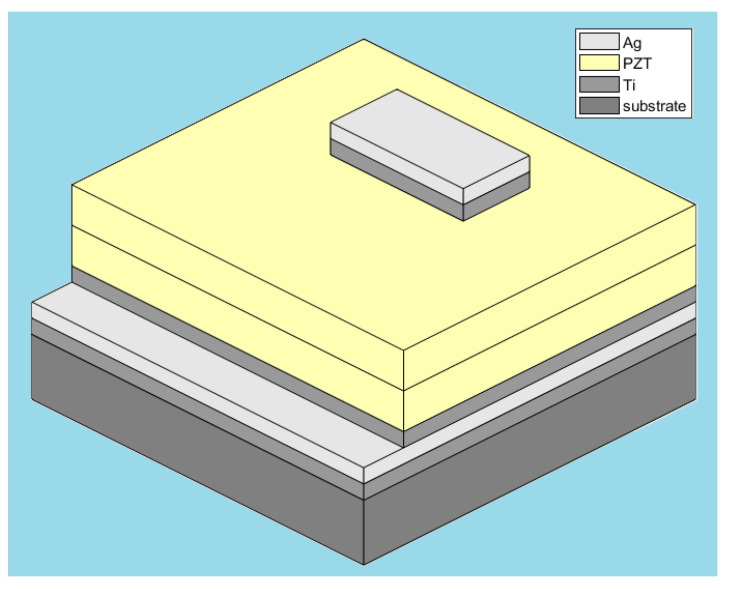
Schematic illustration of the multilayered PZT-based touch sensor structure fabricated in this study.

Based on the characterization results indicating suitability for sensor applications, a multilayered PZT-based touch sensor was fabricated, as depicted schematically in Figure 4.

The device architecture consists of a multilayer stack, where each layer plays a specific structural or functional role. A silicon substrate was used as the base layer. A thin titanium adhesion layer was first deposited to enhance the bonding between the substrate and the silver electrode, which serves as the bottom contact. A titanium layer was applied onto the silver electrode to improve the adhesion of the subsequently deposited PZT layer. This was followed by the deposition of the PZT thin film, acting as the active piezoelectric layer. On top of the PZT film, a titanium layer was again deposited to improve adhesion before forming the top silver electrode. The final silver layer acted as the top contact, completing the metal–ferroelectric–metal (MFM) sensor configuration.

Initially, a thin titanium adhesion layer was deposited onto silicon substrates using DC sputtering; deposition parameters are provided in Table 2. Subsequently, a high-purity silver layer (99.99%) was thermally evaporated onto the titanium layer under a vacuum of approximately $3\times 10^{-6}$ Torr to form the bottom electrode. Approximately 0.2 mg of silver wire, placed in a zirconium crucible, was gradually heated by increasing the current to 125–130 A, evaporating silver onto the titanium surface over roughly 2 min. Then, using the same sputtering conditions, a second titanium layer was created on top of the silver layer.

Following the preparation of this electrode structure, the PZT-1 and PZT-2 layers were sequentially deposited using spin-coating and RF sputtering, respectively. During the spin-coating of the PZT-1 layer, certain regions of the silver electrode were masked using Kapton tape to define the active sensor area. The PZT-2 layer was deposited using RF sputtering through stainless steel shadow masks designed to withstand high temperatures and precisely define film geometry. Finally, top electrodes composed of titanium and silver layers were deposited sequentially using the same masking techniques, completing the multilayer device structure.

**Figure 5:**
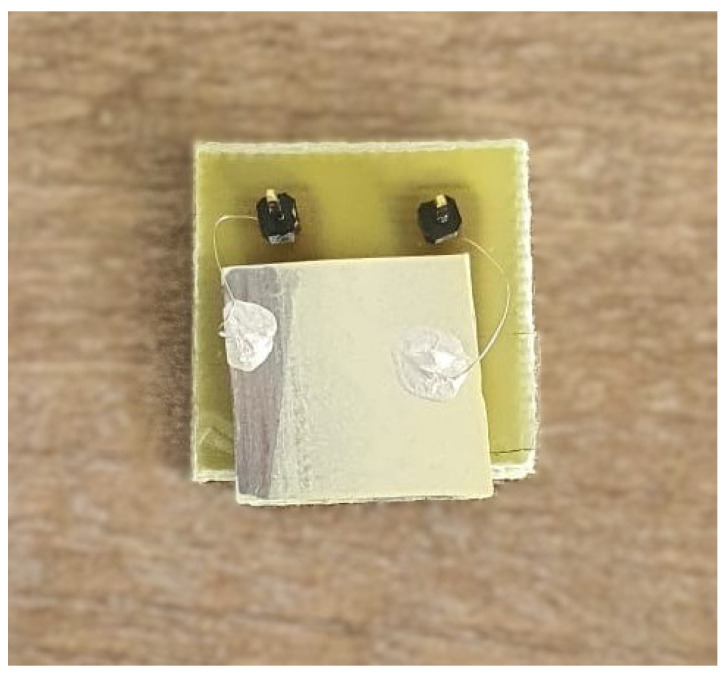
Photograph of the fabricated multilayer PZT-based touch sensor.

Electrical connections to the electrodes were established through wire bonding. AWG-28 wires were bonded to electrode contact pads using silver paste and soldered onto copper terminals. Figure 5 illustrates the final architecture of the device upon the completion of the fabrication process.

## 3. Results and Discussion

**Figure 6:**
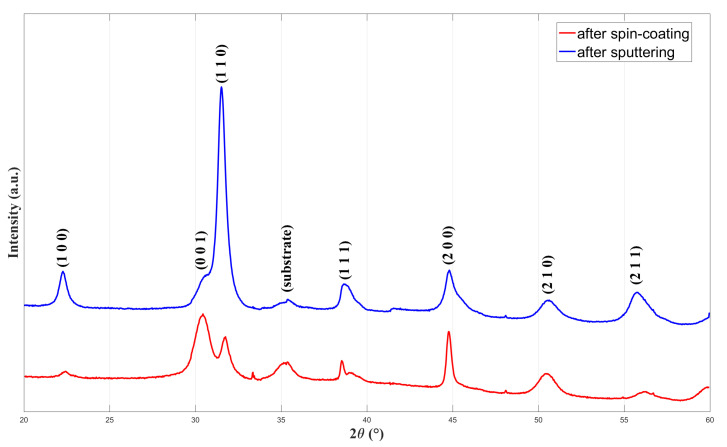
XRD patterns of PZT films after spin-coating and after subsequent RF sputtering.

X-ray diffraction (XRD) analyses were initially conducted to assess the crystalline structure of the deposited PZT thin films, both after the spin-coating process and following the subsequent RF sputtering steps. The XRD results, illustrated in Figure 6, revealed characteristic peaks corresponding to the perovskite phase, specifically identified as the (100), (110), (111), (200), (210), and (211) planes. Minor peaks associated with a secondary pyrochlore phase were also detected, indicating the presence of a low level of impurities. Nevertheless, the dominant phase identified was the desired perovskite structure.

To clarify the impact of the sputtering process, it was observed that the intensities of several diffraction peaks—including (100), (110), (111), and (211)—increased significantly after RF sputtering, suggesting an overall improvement in crystallinity. Additionally, the minor secondary peak near $31^{{}^{\circ}}$ appeared to be suppressed, making the (110) peak more prominent in the pattern. In addition to the higher film thickness, improved orientation alignment and phase purity are responsible for this intensity rise.

Crystallite sizes were estimated using the Scherrer equation by considering all visible diffraction peaks as this approach has been reported to provide more representative average sizes in polycrystalline thin films [32]:

(1) $$D=\frac{K\lambda }{\beta \cos\theta }$$

where *D* is the crystallite size, *K* is the Scherrer constant (typically 0.9), λ is the X-ray wavelength (1.5406 Å), β is the full width at half maximum (FWHM) of the diffraction peak (in radians), and θ is the Bragg angle. The calculated crystallite sizes are presented in Table 3.

**Figure 7:**
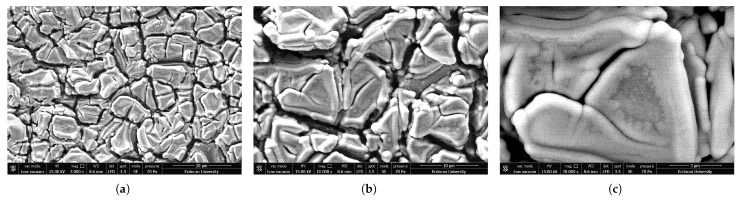
SEM images of PZT film at (**a**) 5000×, (**b**) 10,000×, and (**c**) 30,000× magnification ratios.

Figure 7 displays SEM images taken at different magnifications.

Although SEM images confirmed smooth and homogeneous surface coverage, localized microscopic cracks were observed, likely resulting from internal stresses related to film thickness and post-deposition annealing. These cracks are attributed to internal stresses associated with film thickness and thermal annealing. The literature indicates that similar cracks frequently appear in PZT films exceeding 1 µm in thickness [33].

**Figure 8:**
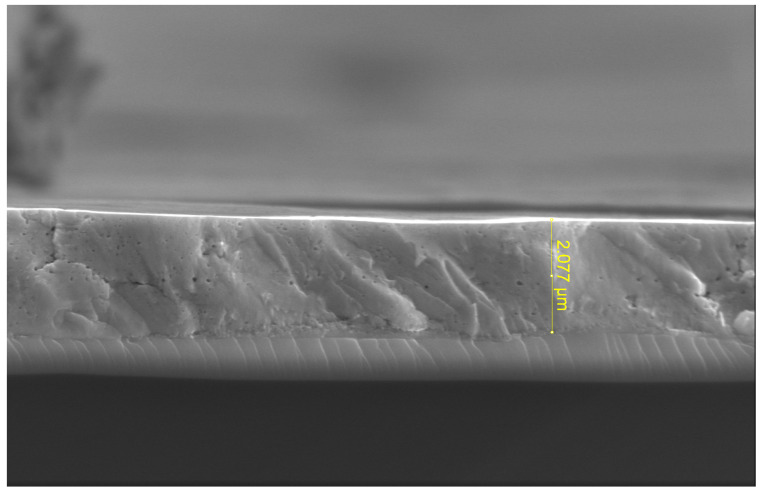
Cross-sectional SEM image of the multilayer PZT film structure, confirming the cumulative thickness obtained through sequential spin-coating and RF sputtering processes.

Cross-sectional SEM analysis in Figure 8 confirmed the total film thickness to be approximately 2 µm, resulting from combined spin-coating and RF sputtering processes.

**Figure 9:**
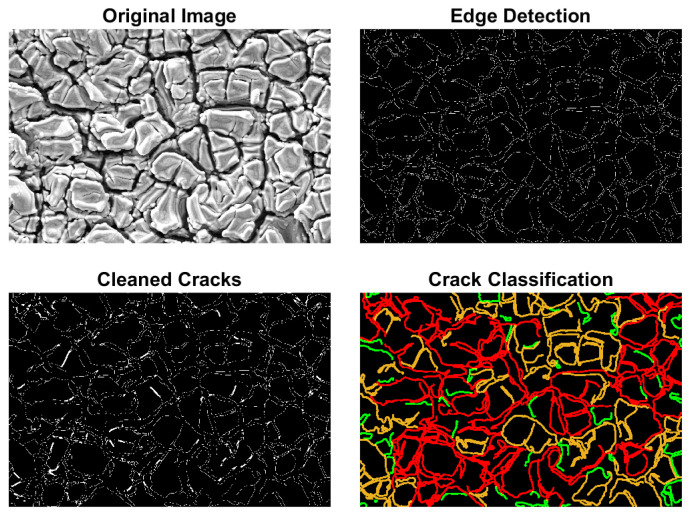
Crack detection and morphological analysis results of the PZT film.

To further analyze the observed cracks, MATLAB 2016b-based image processing was employed. A Gaussian filter (σ=2) was first applied to smooth the images without losing essential details. Subsequently, crack detection was performed using the Canny edge detection algorithm with threshold values of 0.01 (lower) and 0.55 (upper). Detected cracks were classified by size: cracks smaller than 250 pixels were labeled as ‘small’, those larger than 2500 pixels as ‘large’, and those in between as ‘medium’. The resulting processed image, with large cracks marked in red, medium cracks in orange, and small cracks in green, is shown in Figure 9.

**Figure 10:**
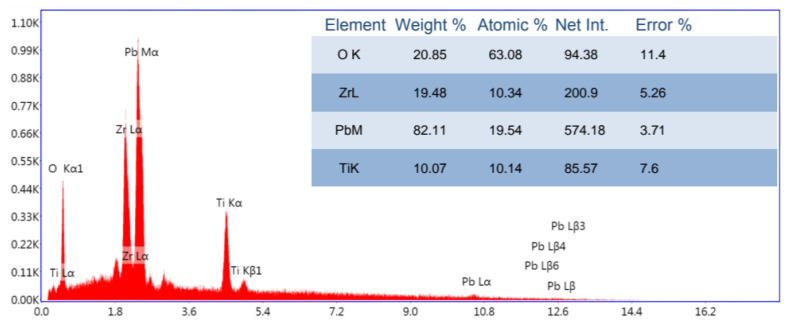
EDX spectrum of the PZT thin film confirming the presence of Pb, Zr, Ti, and O elements.

Energy-dispersive X-ray spectroscopy (EDX) analysis was conducted to confirm the elemental composition of the films, as presented in Figure 10.

The EDX analysis confirmed the successful incorporation of Pb, Zr, Ti, and O into the PZT thin film structure. The measured atomic percentages were in close agreement with the targeted stoichiometry of ${\mathrm{Pb}}_{1}$[${\mathrm{Zr}}_{0.52}$${\mathrm{Ti}}_{0.48}$]$O_{3}$, indicating that the deposition and subsequent annealing processes effectively preserved the intended elemental composition. A minor deficiency in lead (Pb) content was observed, which is a common occurrence in PZT films subjected to high-temperature annealing due to the volatile nature of lead oxides. However, this deviation was within acceptable limits and did not result in the formation of secondary phases or significant alteration of the perovskite structure. Therefore, the EDX results not only validate the phase purity of the film but also demonstrate the reliability of the fabrication process in maintaining near-ideal stoichiometry.

**Figure 11:**
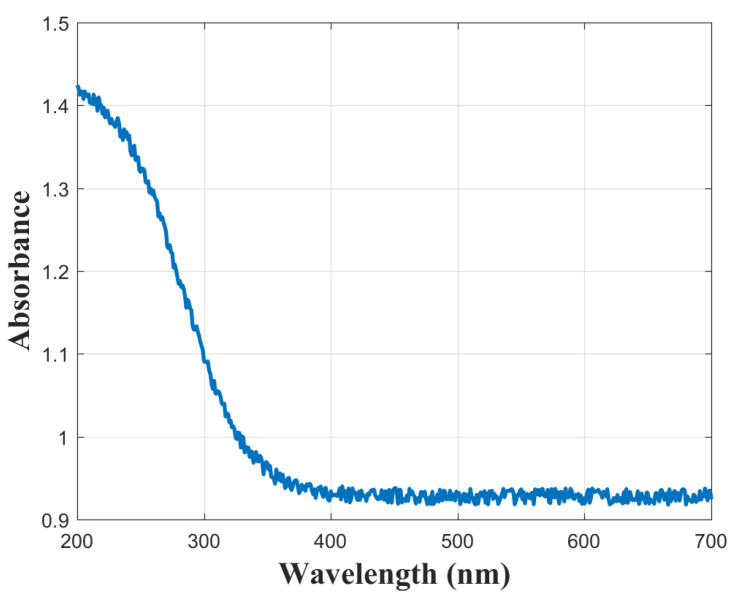
UV–VIS absorbance spectrum of the PZT film.

To investigate the optical properties of the deposited PZT film, the absorbance spectrum recorded by UV–VIS spectroscopy is presented in Figure 11.

**Figure 12:**
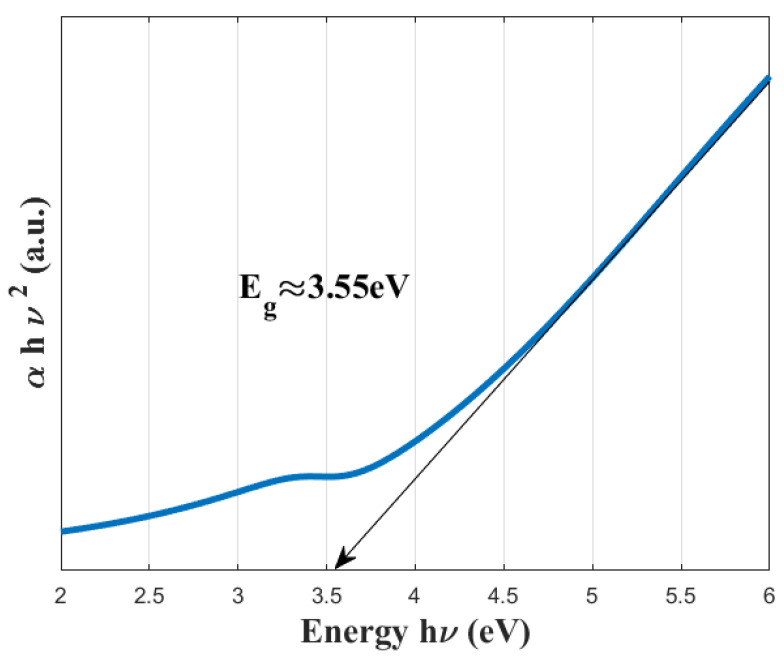
Tauc plot for the band gap estimation of the PZT film.

In addition, the optical band gap was determined using the Tauc plot method applied to the absorbance data presented in Figure 11. The construction of the Tauc plots involved a series of mathematical operations as detailed in Equations (2)–(5).

(2) $$E=\frac{hc}{\lambda }$$

where *h* is Planck’s constant $\left(4.1357\times 10^{-15}\;\mathrm{eV}\cdot s\right)$, *c* is the speed of light $\left(3\times 10^{17}\;\mathrm{nm}/s\right)$, and λ is the wavelength in nanometers. By substituting the constants into the equation, it simplifies to Equation (3):

(3) $$E=\frac{1241}{\lambda }$$

which provides the *x*-axis values of the Tauc plot. To obtain the absorption coefficient α, Equation (4) was used:

(4) $$\alpha =\frac{A}{d}$$

where *A* is the absorbance obtained from measurements and *d* is the film thickness in centimeters. These α values were then used in Equation (5) to compute the *y*-axis values of the Tauc plot:

(5) $${\left(\alpha h\nu \right)}^{2}=B\left(h\nu -E_{g}\right)$$

where *B* is a material-dependent constant known as the Tauc constant, $E_{g}$ is the optical band gap, and hν is the photon energy. The linear portion of the resulting plot was extrapolated to determine the optical band gap of the films. Figure 12 depicts the Tauc plots constructed based on the aforementioned equations.

By extrapolating the linear region of the graph to the *x*-axis, the optical band gap was determined to be approximately 3.55 eV, confirming the agreement of the films with the literature [34].

**Figure 13:**
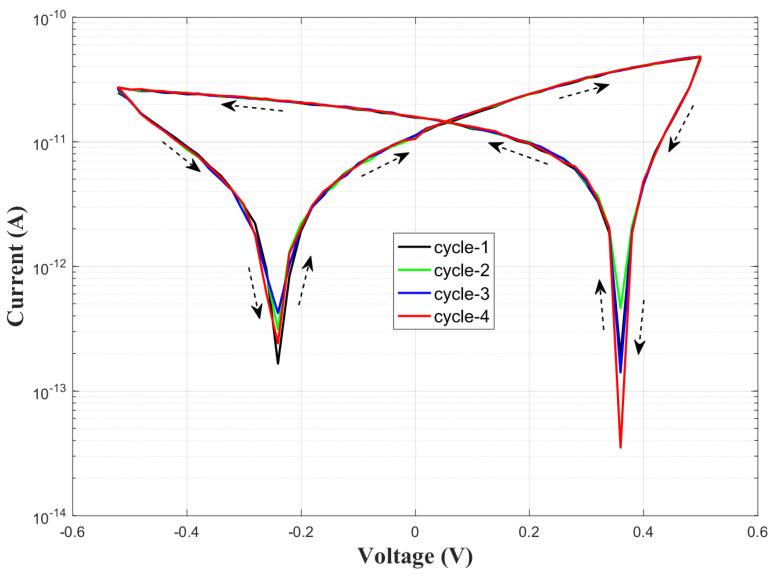
Current–voltage (I–V) characteristics of the PZT-based sensor measured via cyclic voltammetry in the ±0.5 V range.

Given these favorable results, a PZT-based touch sensor was subsequently designed and fabricated. The current–voltage (I–V) characteristics, measured via cyclic voltammetry within the ±0.5 V range, are shown in Figure 13.

**Figure 14:**
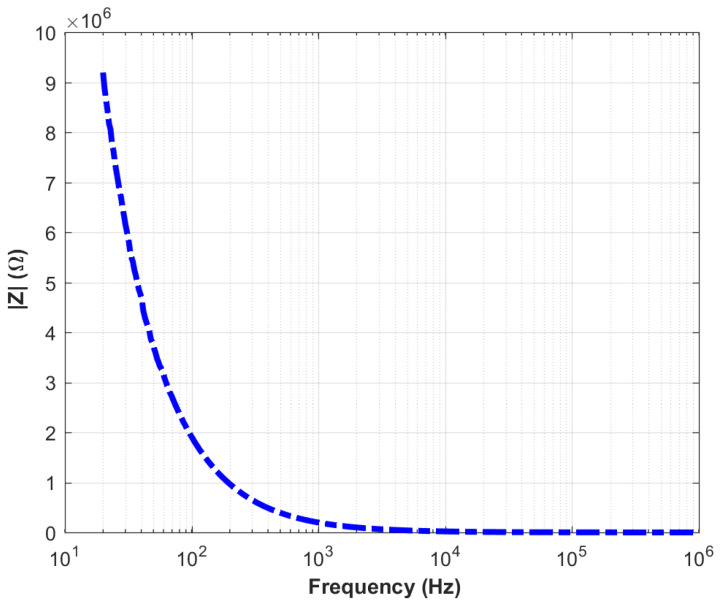
Frequency-dependent impedance characteristics of the PZT sensor measured in the 100 Hz to 1 MHz range.

A progressive increase in current was observed across successive voltage cycles, suggesting enhanced ferroelectric polarization and improved mobility of charge carriers within the PZT structure. The hysteresis observed in the I–V curves demonstrates the reversible electrical behavior of the piezoelectric material, underscoring the sensor’s repeatability and stability. Impedance spectroscopy was conducted to determine the sensor’s resonance characteristics, with the frequency-dependent impedance (Z–f) results depicted in Figure 14.

**Figure 15:**
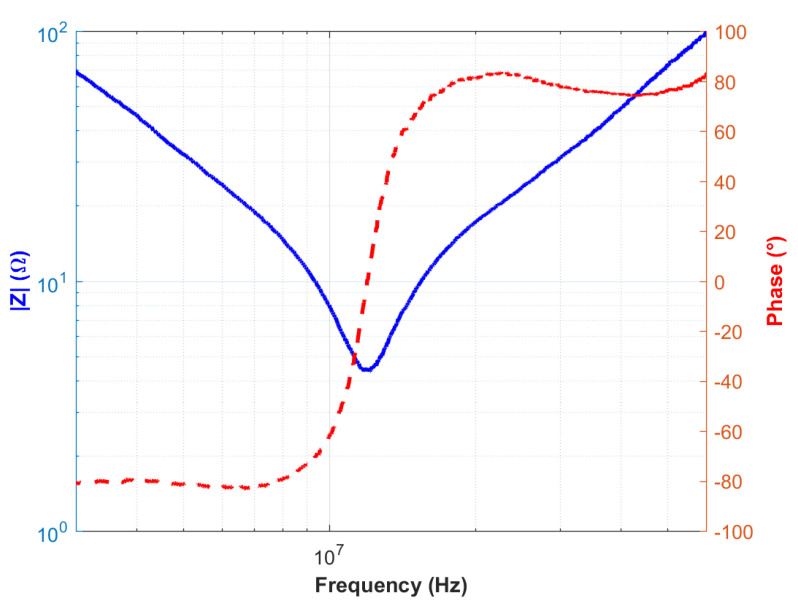
Frequency-dependent impedance magnitude and phase angle response of the fabricated PZT sensor measured in the 1–100 MHz range.

When Figure 14 is examined, the expected dielectric behavior is observed at low frequencies. However, no resonance or anti-resonance phenomena are detected within the measured frequency range. Therefore, impedance measurements at higher frequencies were conducted. The impedance versus frequency graph obtained from measurements performed with a vector network analyzer in the range of 1 MHz to 100 MHz is presented in Figure 15.

**Figure 16:**
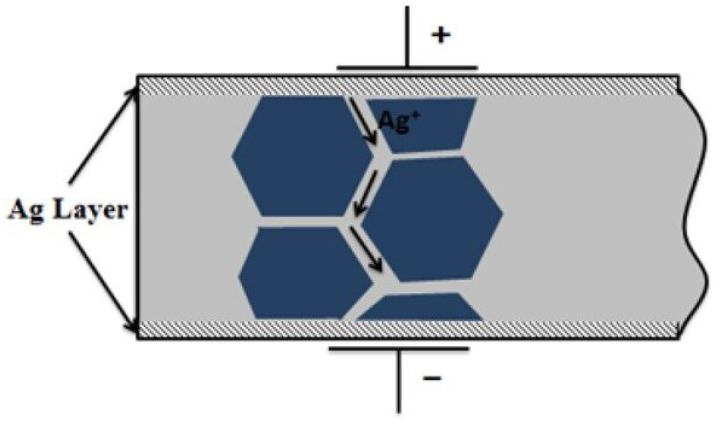
Schematic representation of silver diffusion into the PZT structure, leading to high-frequency inductive anomalies [35].

The sensor exhibited resonance behavior at approximately 12 MHz, evidenced by a minimum impedance and a significant phase shift. However, an expected anti-resonance peak was not observed, likely due to degradation effects within the piezoelectric structure [35,36]. As shown in Figure 15, After the phase angle transitions into the positive region—indicating inductive behavior—it fails to return to a capacitive response, and the expected sharp impedance peak is not observed. This phenomenon is believed to be associated with silver diffusion from the electrode layers into the PZT structure. The diffused silver ions are thought to form conductive pathways that introduce parallel inductive effects, thereby maintaining the sensor’s response in the inductive regime. Such diffusion-induced effects can degrade sensor performance at high frequencies. Nevertheless, the cyclic voltammetry results maintained excellent reproducibility and symmetrical hysteresis, indicating robust electrical stability at lower frequencies. Figure 16 schematically illustrates the proposed migration pathways of silver ions within the PZT layer, which are believed to be responsible for the observed high-frequency phase anomalies.

**Figure 17:**
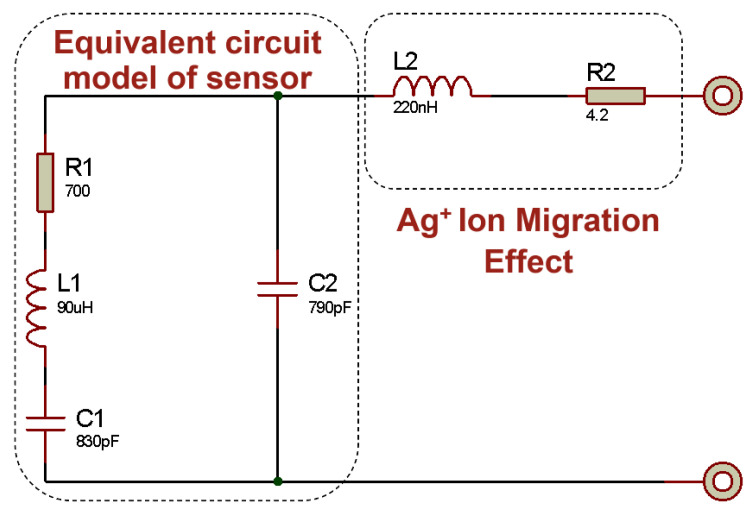
Equivalent circuit model of the PZT sensor, including additional elements representing degradation effects caused by ${\mathrm{Ag}}^{+}$ ion migration at high frequencies.

To quantitatively analyze these anomalies, an electrical equivalent circuit of the sensor structure was developed. This equivalent circuit models the piezoelectric response of the sensor and its behavior at high frequencies, explaining impedance variations associated with both resonance and anti-resonance frequencies. The circuit primarily consists of a series-connected RLC branch and a parallel dielectric capacitance ($C_{0}$). The series RLC element electrically represents the mechanical resonance of the piezoelectric material, while the parallel capacitance $C_{0}$ accounts for the geometric capacitance arising from the electrodes and substrate. This configuration is commonly known as the Butterworth–Van Dyke model [37]. Furthermore, the abnormal phase shifts and impedance increases observed at high frequencies in some measurements can be explained by augmenting this model with an additional inductive element and a low-value conductive resistance in parallel. The added inductance represents conductive pathways within the structure, analogous to that depicted in Figure 16, which may result from diffusion or leakage currents originating from the silver electrodes. Consequently, the model is extended to capture not only the ideal resonance response but also electrical manifestations of physical phenomena such as structural distortions, ion migration, and interfacial diffusion. The schematic of the equivalent circuit described above is shown in Figure 17.

**Figure 18:**
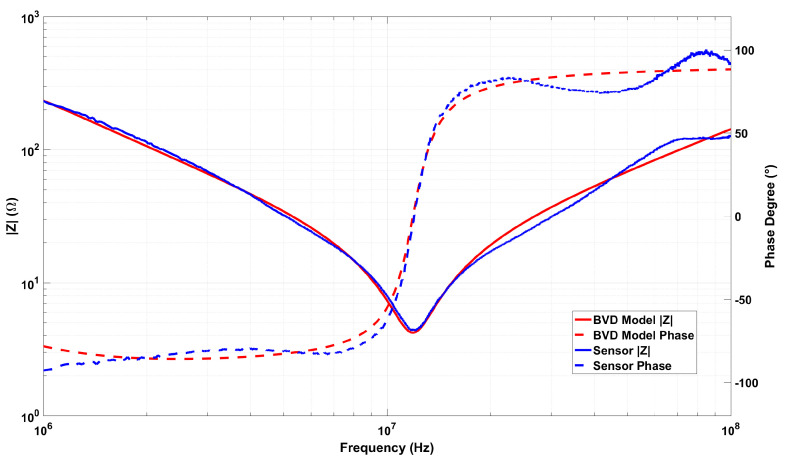
Frequency-dependent variations in the impedance and phase angle of the sensor structure along with the corresponding BVD circuit simulation results.

As a result, the theoretical impedance and phase responses derived from the equivalent circuit model were compared with the experimental measurements, demonstrating excellent agreement. The corresponding illustration is shown in Figure 18.

Upon examination of Figure 18, it is evident that the measurement and simulation results exhibit closely matching curves. This correspondence indicates that the equivalent circuit accurately represents the sensor structure. The parameter values of $L_{1}$, $C_{1}$, $R_{1}$, and $C_{0}$ in the equivalent circuit are further validated by series and parallel measurements obtained from the LCR meter. The elements $L_{2}$ and $R_{2}$, which have been modified to closely match the experimental data, are linked to silver ion migration. Maintaining a very low value for $R_{2}$ aligns with the expected behavior of ion diffusion. Nevertheless, the observed fluctuations around 50 MHz and beyond can be attributed to alternative leakage pathways within the device structure.

Controlled touch stimulation experiments, involving repeated finger touches, were performed to evaluate the practical performance of the fabricated sensor. To facilitate accurate detection and analysis of the sensor’s inherently low-amplitude piezoelectric output signals, a dedicated instrumentation amplifier was designed and implemented. The amplifier circuit was optimized to ensure high input impedance, minimal noise, and differential signal processing capabilities. Detailed schematics and the printed circuit board layout of the amplifier are provided in the Supplementary Materials.

**Figure 19:**
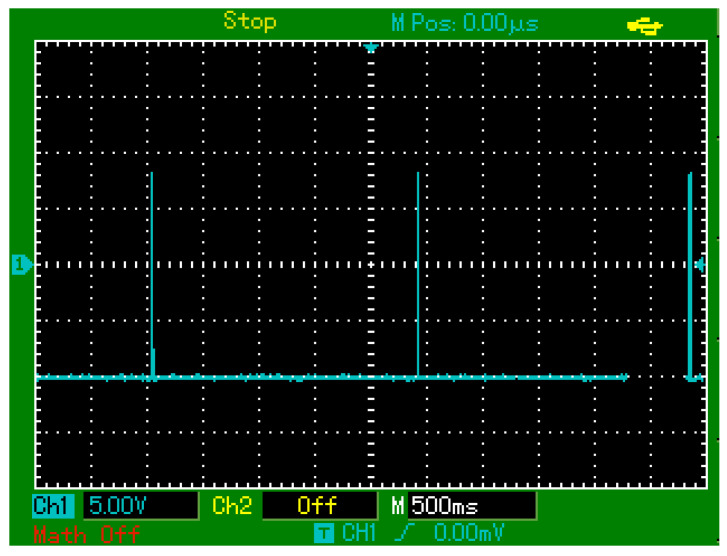
Oscilloscope-recorded output signals of the PZT-based sensor in response to periodic finger touches applied at approximately 2.5 s intervals.

Controlled touch stimulation experiments involving repeated finger touches were performed to characterize the sensor’s practical performance. Initial tests with touches at approximately 2.5 s intervals produced distinct, repeatable electrical responses, as recorded by oscilloscope data (Figure 19).

**Figure 20:**
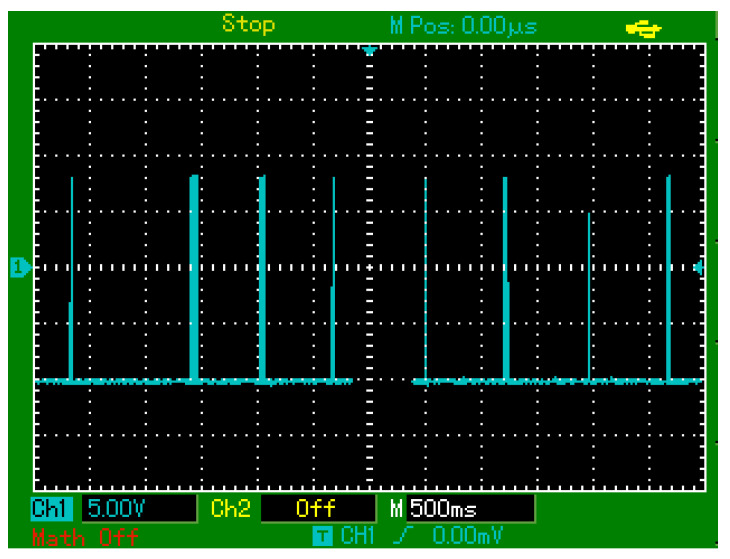
Oscilloscope-recorded output signals of the PZT-based sensor in response to periodic finger touches applied at approximately 0.5 s intervals.

In subsequent tests with higher-frequency touches (approximately 0.5 s intervals), most touches were successfully detected; however, about 10% were missed (Figure 20), indicating limitations in response time or signal processing.

**Figure 21:**
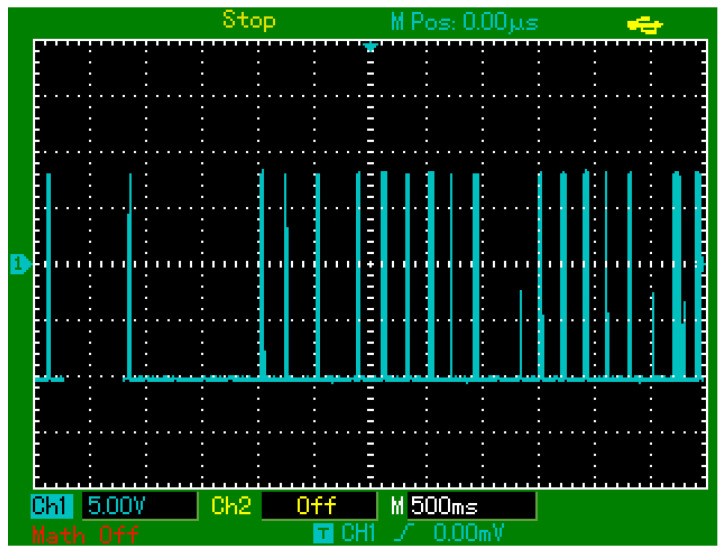
Oscilloscope-recorded output signals of the PZT-based sensor in response to randomly timed finger touches under realistic usage conditions.

Lastly, random touch tests under realistic, irregular timing conditions demonstrated the sensor’s effectiveness, with consistent responses and no false signals or distortions, as shown in Figure 21.

It is important to note that the current setup does not include a calibrated mechanical loading system capable of applying known and repeatable force values. As a result, a direct force–voltage relationship could not be established in this study. Nevertheless, the sensor consistently produced reproducible voltage outputs in response to mechanical contact, as confirmed by oscilloscope measurements. These results demonstrate the functional piezoelectric response of the device under real-world touch conditions.

## 4. Conclusions

In this study, PZT thin films were successfully fabricated using a hybrid deposition approach combining spin-coating and RF sputtering techniques. This two-step process enabled the formation of high-quality films with desirable structural and functional properties. X-ray diffraction (XRD) analyses confirmed the successful formation of the target perovskite crystal structure in all samples. Notably, samples subjected to the additional sputtering step exhibited significantly enhanced phase purity and crystallinity, as evidenced by sharper and more intense diffraction peaks. Crystallite sizes calculated via the Scherrer equation ranged from approximately 7.43 nm to 16.52 nm, indicating the presence of well-ordered nanostructures within the films.

Scanning electron microscopy (SEM) was employed to analyze the surface morphology and assess the overall integrity of the PZT films. The analyses revealed that the samples possessed relatively smooth and homogeneous surfaces, indicating good film formation and adhesion to the substrate. However, microscopic cracks were observed in certain regions, which were attributed to internal stress accumulation during the drying and annealing stages. These stresses are typically associated with thickness gradients, thermal expansion mismatch, and the rapid volatilization of organic components during the spin-coating process. The presence of cracks was further confirmed through MATLAB-based image processing.

The optical properties of the PZT films were evaluated via UV–VIS spectroscopy. Based on absorbance measurements, the optical band gap was determined using the Tauc plot method to be approximately 3.55 eV. This wide band gap suggests that the fabricated films are well-suited for applications in optoelectronics, piezo-optoelectronic sensors, and other high-frequency or light-sensitive electronic systems. Moreover, the low optical absorption in the visible range implied by this high band gap makes these films advantageous for use in transparent or photosensitive device architectures.

Capitalizing on these promising material characteristics, a touch sensor was developed by integrating the PZT film structure with electrode configurations. The sensor’s I–V characteristics demonstrated remarkable repeatability. In addition to DC response analysis, impedance–frequency (Z–f) measurements were conducted to investigate the sensor’s frequency-dependent electrical behavior. While the measurements revealed the expected dielectric response at low frequencies, no distinct anti-resonance peaks were observed at higher frequencies. This absence of anti-resonance was attributed to degradation mechanisms likely induced by silver (${\mathrm{Ag}}^{+}$) ion diffusion from the electrode layer into the PZT structure.

To account for these non-idealities, the classical Butterworth–Van Dyke (BVD) equivalent circuit model was extended by incorporating additional parasitic elements. Specifically, a parallel inductive branch and a low-resistance leakage path were added to simulate the effects of internal conductive pathways arising from ${\mathrm{Ag}}^{+}$ ion migration. This extended model not only improved the fitting accuracy with the experimental Z–f data but also provided insight into the physical mechanisms responsible for the observed deviations from ideal piezoelectric resonance behavior.

The functional performance of the sensor was assessed through systematic experiments. Oscilloscope measurements demonstrated that the device could detect mechanical stimuli, such as finger touches, with high sensitivity and repeatability. With periodic stimuli applied at approximately 2.5 s intervals, the sensor produced clear and consistent output signals for each contact event. Even with faster stimuli applied at 0.5 s intervals, the sensor maintained a detection accuracy of approximately 90%, missing only a small fraction of touches. During random timing tests, the sensor responded accurately and reliably to each contact, demonstrating sufficient stability and sensitivity under realistic operating conditions.

Although the device demonstrated reliable electrical output under mechanical stimulation conditions, a quantitative force-resolved analysis could not be performed due to the lack of a calibrated force application system. Future work will focus on integrating such a system to enable precise measurement of output characteristics such as voltage-per-force sensitivity, response time, and dynamic behavior.

Overall, the structural, morphological, optical, electrical, and functional evaluations strongly suggest that the developed PZT-based film structure is a promising candidate for touch-sensing applications. It offers an attractive combination of high crystallinity, surface uniformity, optical transparency, and robust electrical response. Future studies will aim to mitigate the observed micro-cracks using advanced stress-relief strategies or optimized thermal treatments. Additionally, efforts will be directed toward modeling the suppression of anti-resonance behavior observed in silver–silver electrode configurations.

## Figures and Tables

**Table 1:** Deposition parameters used in the RF sputtering of PZT thin films.

Parameter	Value
Initial Pressure	$5\times 10^{-6}$ Torr
RF Power	100 W
Working Pressure	5 mTorr
Argon Flow Rate	25 sccm
Substrate Temperature	${300\;}^{{}^{\circ}}$C
Deposition Time	30 min

**Table 2:** Deposition parameters for the DC sputtering of titanium adhesion layers.

Parameter	Value
Initial Pressure	$5\times 10^{-6}$ Torr
DC Power	100 W
Working Pressure	10 mTorr
Argon Flow Rate	20 sccm
Target-Substrate Distance	80 mm
Deposition Time	5 min

**Table 3:** Crystallite sizes of PZT thin films calculated using the Scherrer equation.

Miller Indices	2θ (°)	β (°)	D (nm)	Average D (nm)
(100)	22.27	0.49	16.52	12.15
(110)	31.52	0.56	14.73	
(111)	38.66	0.71	11.79	
(200)	44.81	0.62	13.95	
(210)	50.60	1.03	8.53	
(211)	55.75	1.21	7.43	

## Data Availability

The original contributions presented in this study are included in the article/Supplementary Material.

## Supplementary Materials

The following supporting information can be downloaded at: https://www.mdpi.com/article/10.3390/s25133938/s1, Figure S1: Schematic diagram of the differential amplifier circuit used to amplify the output of the PZT-based touch sensor; Figure S2: Photograph of the fabricated amplifier circuit implemented on a prototype board corresponding to the schematic in Figure S1.
