# Supplementary figures and images for: Fabrication and Characterization of a PZT-Based Touch Sensor Using Combined Spin-Coating and Sputtering Methods
Source: Sensors (Basel). 2025 Jun 24;25(13):3938. doi: 10.3390/s25133938 (PMC12252296; doi:10.3390/s25133938)

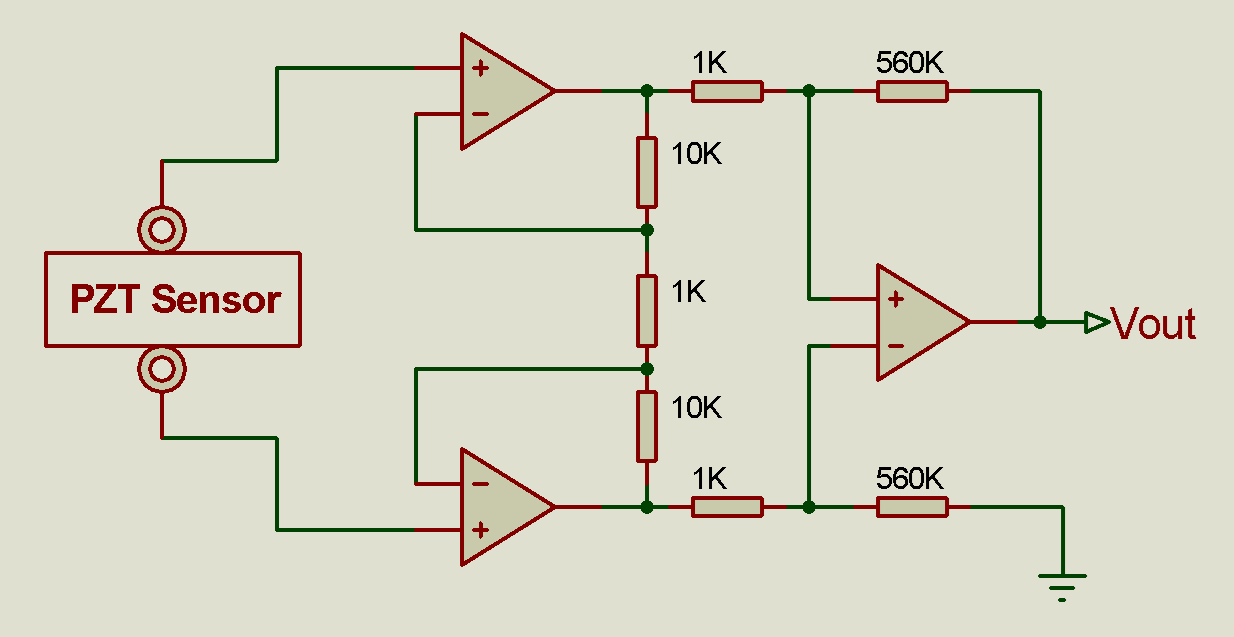

Supplement: Supplementary file 1 [file sensors-25-03938-s001.zip › Figure S1.png]

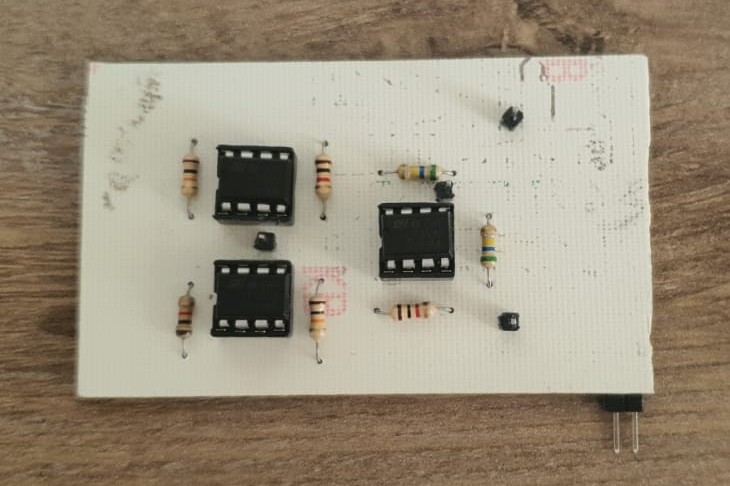

Supplement: Supplementary file 1 [file sensors-25-03938-s001.zip › Figure S2.jpeg]
